# Atrial Tram Tracks and Ventricular Step Ladder: Decoding the Dot Plot

**DOI:** 10.1002/joa3.70266

**Published:** 2026-01-09

**Authors:** Ramanathan Velayutham, Anish Bhargav, Barathkrishnan Janarthanan, Raja J. Selvaraj

**Affiliations:** ^1^ Department of Cardiology Jawaharlal Institute of Postgraduate Medical Education and Research Puducherry India

## Abstract

Falsely detected atrial tachycardia episode in a patient with CRT‐P due to FFRW oversensing resulting in ventricular sensed response triggered BiV pacing and auto adjusting sensitivity phenomenon.

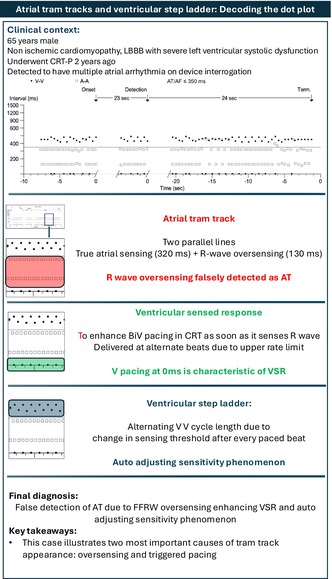

**Abbreviations**
CRT: cardiac resynchronization therapy
EGM: electrogram
VSR: ventricular sensed response

A 65‐year‐old male underwent implantation of a biventricular cardiac resynchronization therapy pacemaker (bipolar CRT‐P device, Solara CRT‐P W1TR06 from Medtronic) 8 years ago for non‐ischemic cardiomyopathy with left bundle branch block and severe left ventricular systolic dysfunction. On his routine device clinic follow up, device interrogation showed an arrhythmia episode detected as atrial tachycardia. Dot plot of this episode is shown below (Figure 1). Black dots represent V event, and white squares represent atrial events. Figure 2 shows detail of one segment of the dot plot with the corresponding electrograms. What does this represent?

**FIGURE 1:**
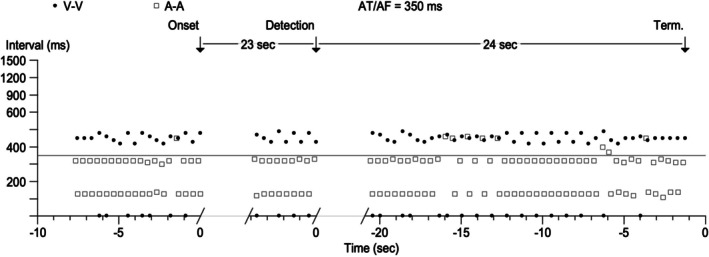
Dot plot tracing of device detected atrial tachycardia event in a patient with CRT‐P. Black dots represent V event and white squares represent atrial events.

**FIGURE 2:**
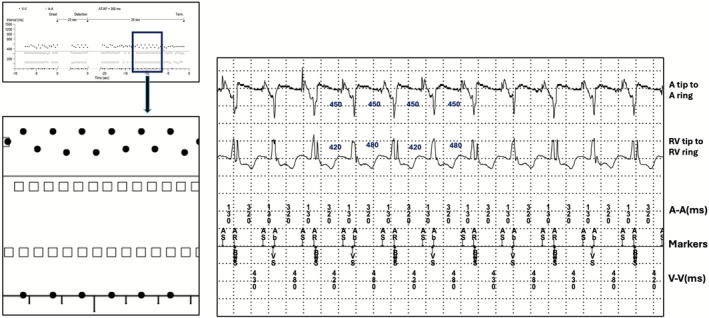
Zoomed inset of the dot plot with corresponding EGM. The ventricular marker channel denotes Vs for ventricular sensed events and BV overlaps with VS whenever there is Bi V pacing along with ventricular sensed events. AS, AR, and AB in the atrial marker channel represent atrial sensed, atrial refractory, and atrial blanked events respectively.

## 1 Discussion

Atrial dot plot pattern shows two parallel tram lines at cycle length of 320 ms and 130 ms. This pattern is typical of R wave oversensing and this is confirmed from the electrograms. The true atrial cycle length is derived as the sum of these two cycle lengths which is 450 ms (133 bpm).

Analyzing the segment of ventricular dot plot shown in Figure 2, ventricular events are seen at two cycle lengths of 420 and 480 ms. The average of these equals the true atrial cycle length of 450 ms suggesting that these represent true sensing. In addition, there is a ventricular event happening every alternate beat at cycle length of 0 ms. Ventricular sensing at this interval of 0 ms suggests ventricular sensed response(VSR) and this is confirmed in the EGM (marked as BV) every alternate beat. Ventricular sense response is a feature of CRT to enhance biventricular pacing. It works both in non‐tracking and tracking mode. In nontracking mode, a sensed right ventricular event below the maximum tracking rate triggers an immediate ventricular pacing pulse. In tracking mode, a sensed right ventricular event during the AV interval triggers an immediate ventricular pacing pulse. As this patient was detected to have atrial tachycardia due to R wave oversensing, the device has switched to non‐tracking mode and thereby whenever a ventricular event is sensed below the maximum tracking rate i.e., coupling interval > 460 ms, it triggers biventricular pacing.

**FIGURE 3:**
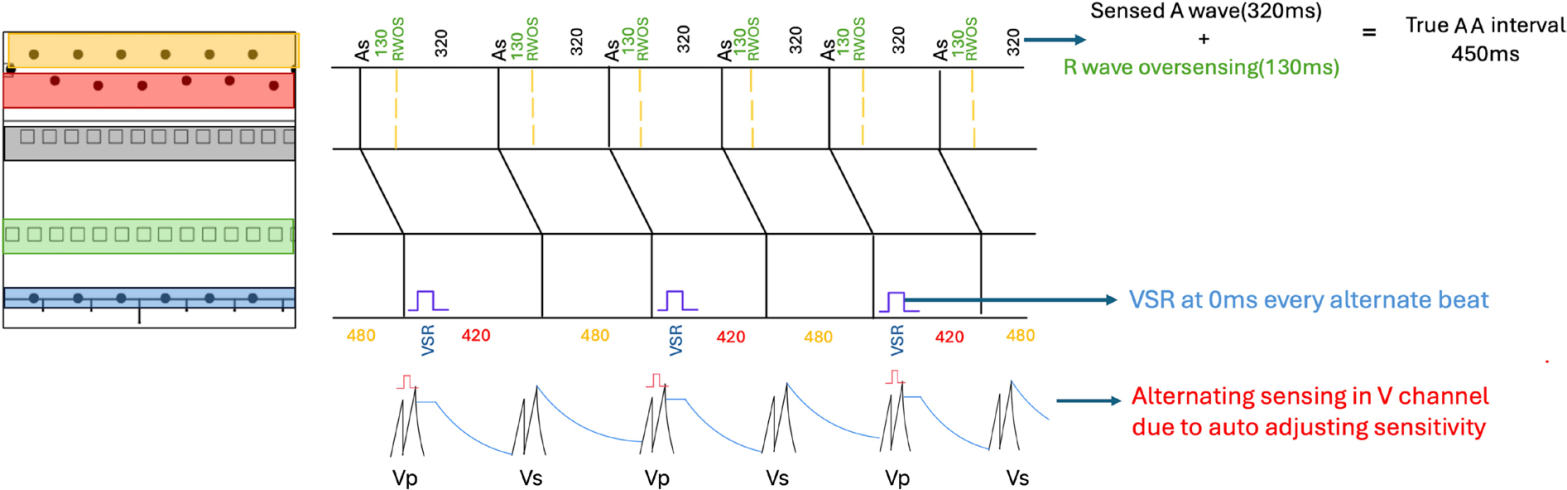
Dot plot with ladder diagram depiction. Atrial EGM with black shaded events represents true atrial activity. Atrial events shaded with green represent R wave oversensing at 130 ms after atrial event. The true atrial cycle length is therefore 320 ms + 130 ms which is 430 ms. The lower blue shaded ventricular events at 0 ms every alternate beat represents ventricular sensed events triggering Biventricular pacing by ventricular sensed response(VSR). VSR happens at every alternate beats only when the sensed events are below the maximum tracking rate. Ventricular events at red and orange represents variation in cycle length every alternate beats which is explained by auto adjusting sensitivity phenomenon.

From the ventricular EGM analysis it is evident that there is periodic variation in ventricular cycle length at 420 ms and 480 ms. On closer inspection it is seen that the far field R wave sensed in atrial channel showed a fixed CL of 450 ms. This periodic variation of shorter cycle length in near field EGM after biventricular pacing is due to variation in the timing at which sensing occurs because of the auto adjusting sensitivity phenomenon. The auto adjusting sensitivity phenomenon varies the sensitivity after ventricular paced and sensed events to enhance better R wave sensing. Though the auto adjusting sensitivity phenomenon is a key feature of most ICD devices and also few CRT devices such as Solara devices. After a ventricular sensed event, the device automatically adjusts sensitivity to thresholds to 10 times the programmed value. After a ventricular paced event, the threshold is set at 4.5 times the programmed value and there is a blanking period of 200 ms. Both these factors contribute to variation at the time at which the R wave is sensed in near field EGM (Figure 3).

This case illustrates the two most common causes of tram track appearance in the form of oversensing (R wave oversensing in the atrial channel) and triggered pacing (ventricular sensed response). Dot plot at cycle length of 0 ms in CRT is characteristic of ventricular sensed response. Dot plot of alternating beats at closer cycle length suggests variation in threshold of sensing at near field channel.

## Funding

The authors have nothing to report.

## Ethics Statement

Written and informed patient consent obtained.

## Conflicts of Interest

The authors declare no conflicts of interest.

## Data Availability

The authors have nothing to report.

